# The Application of Near-Infrared Spectroscopy Combined with Chemometrics in the Determination of the Nutrient Composition in Chinese *Cyperus esculentus* L.

**DOI:** 10.3390/foods14030366

**Published:** 2025-01-23

**Authors:** Xiaobo Jiao, Dongliang Guo, Xinjun Zhang, Yunpeng Su, Rong Ma, Lewen Chen, Kun Tian, Jingyu Su, Tangnuer Sahati, Xiahenazi Aierkenjiang, Jingjing Xia, Liqiong Xie

**Affiliations:** 1 Xinjiang Key Laboratory of Biological Resources and Genetic Engineering, College of Life Science and Technology, Xinjiang University, Urumqi 830046, China; jxb@stu.xju.edu.cn (X.J.);; 2 College of Smart Agriculture, Xinjiang University, Urumqi 830046, China

**Keywords:** near-infrared spectroscopy, tiger nut, nutritional content, variable selection algorithms

## Abstract

The nutritional content of tiger nut (*Cyperus esculentus* L.) is abundant, rich in oil, protein, and starch. Conventional methods for assessing the nutrient composition of tiger nuts (TNs) are time-consuming and labor-intensive. Near-infrared spectroscopy (NIR) combined with chemometrics has been widely applied in rapidly predicting the nutritional content of various crops, but its application to TNs is rare. In order to enhance the practicality of the method, this study employed a portable NIR in conjunction with chemometrics to rapidly predict the contents of crude oil (CO), crude protein (CP), and total starch (TS) from TNs. In the period from 2022 to 2023, we collected a total of 75 TN tuber samples of 28 varieties from Xinjiang Uyghur Autonomous Region and Henan Province. The three main components were measured using common chemical analysis methods. Partial least squares regression (PLSR) was utilized to establish prediction models between NIR and chemical indicators. In addition, to further enhance the prediction performance of the models, various preprocessing and variable selection algorithms were utilized to optimize the prediction models. The optimal models for CO, CP, and TS exhibited coefficient of determination (R^2^) values of 0.8946, 0.8525, and 0.8778, with root mean square error of prediction (RMSEP) values of 1.1764, 0.7470, and 1.4601, respectively. The absolute errors between the predicted and actual values for the three-indicator spectral measurements were 0.80, 0.59, and 0.99. The results demonstrated that the portable NIR combined with chemometrics could be effectively utilized for the rapid analysis of quality-related components in TNs. With further refinements, this approach could revolutionize TN quality assessment and be used to determine optimal harvest times, as well as facilitate the graded marketing of TNs.

## 1. Introduction

The tuber of tiger nuts (*Cyperus esculentus* L.) is rich in nutrients, earning it the title of the “King of Oil Crops”. Recent investigations have shown that tiger nuts (TNs) are high in oil content (17–35.43%), and rich in monounsaturated fatty acids, polyphenols, tocopherols, and phytosterols, as well as high-value compounds such as proteins (5–9.7%), carbohydrates (16.2–47%), vitamins (Vitamin A, C, D, and E), minerals, and bioactive compounds [1]. The TN can be used for oil extraction, brewing, and other economically valuable applications, and is well-suited for growth in sandy soils, significantly enhancing the utilization of marginal lands. The oil extracted from TNs shares similarities with olive oil, both of which contain over 70% monounsaturated fatty acids and are rich in polyphenols, tocopherols, and phytosterols [1,2]. The starch from TNs exhibits good resistance to digestion, which is beneficial for lowering blood sugar levels [3]. The proteins in TNs are rich in essential amino acids, particularly lysine, threonine, and leucine [4]. They exhibit high digestibility and favorable nutritional properties, making TN an excellent source of plant-based protein [5]. Since these indicators are crucial for assessing the quality and nutritional value of TNs, providing scientific evidence and guidance for its cultivation, processing, and utilization, there is an urgent need for an efficient detection method to measure these indicators.

Researchers often use conventional chemical analysis methods in the laboratory to detect the chemical components in TNs. For example, protein content is commonly determined using the Kjeldahl method [6] or the Bicinchoninic Acid (BCA) assay [5]. Oil content is measured using the Soxhlet extraction method [6], gas chromatography [7], or liquid chromatography [8], and total starch content is analyzed using a dual-wavelength colorimetric method [3,9]. However, compared to the seeds of crops such as wheat and rapeseed, TNs are larger, and the small sample sizes used in chemical analysis methods may not be representative, failing to accurately reflect the distribution of the chemical components in the sample population. Additionally, chemical analysis is time-consuming and labor-intensive, making it unsuitable for large-scale and rapid quality analysis. Moreover, destructive analysis methods are not convenient for subsequent breeding and variety improvement. Therefore, to meet the systematic evaluation needs of TNs from different regions, cultivation conditions, and varieties, it is necessary to establish a non-destructive and rapid detection system.

There are an increasing number of technologies that involve near-infrared (NIR) light [10]. NIR spectroscopy offers the advantages of speed, non-destructiveness, and multi-component analysis, making it widely used in agriculture [11], food [12], pharmaceuticals [13], petrochemicals [14], and other fields. NIRS utilizes multiple wavelengths of NIR light to retrieve the material information of an object, especially its chemical components [10]. NIR measures the absorption intensity of samples at different wavelengths of near-infrared light to obtain the near-infrared spectrum. NIR primarily records the overtone and combined absorption of vibrations from hydrogen-containing groups X–H (where X = C, N, O), making it applicable to agricultural products for quality assessment, variety identification, and other analytical purposes [15]. NIR technology enables the rapid and non-destructive determination of the typical quality characteristics of food categories [16]. Hence, NIRS is superior for mobile material sensing tasks, such as identifying food compositions, water quality analysis, and crop disease detection [10]. For example, Tarandeep Singh et al. used a convolutional neural network (CNN) to determine the protein content in barley samples, achieving a coefficient of determination (R^2^) of 0.9962 and a root mean square error prediction (RMSEP) of 0.0823, allowing for the accurate prediction of the protein content in barley [17]. Özcan Çataltas et al. utilized a one-dimensional convolutional autoencoder combined with NIR to detect protein, starch, oil, and moisture content in corn kernels. The R^2^ values for these were 0.9012, 0.9359, 0.9632, and 0.9716, respectively, with corresponding RMSEP values of 0.1535, 0.2093, 0.0388, and 0.0628 [18]. Edenio Olivares et al. employed logistic regression, support vector machine (SVM), and partial least squares discriminant analysis (PLSDA) models to predict rice protein and amylose content, achieving a high accuracy of 94%, an F1-score of 90%, an average precision of 94%, and a low classification error rate of 4%. This enabled the accurate and non-destructive classification of rice quality [19]. NIR has also made significant contributions to the quantitative analysis of soybean proteins, oils, and fatty acids [20,21,22]. However, there are no relevant articles on near-infrared analytical models for TNs. Therefore, this paper selected NIR spectroscopy, combined with chemometric methods, to establish predictive models for nutritional indicators in TNs. In cereal composition analysis, the rapid detection of oil, protein, and starch using NIR is well established. Consequently, the application of NIR for the analysis of TN components holds potential and is feasible.

Commonly used modeling algorithms, such as partial least squares regression (PLSR) [23] and principal component regression (PCR) [24], typically perform full-spectrum analysis. However, in NIR, the number of variables frequently far exceeds the number of samples. Consequently, collinearity and redundancy are common phenomena in the data matrix, and irrelevant information such as background, noise, and overlapping bands can affect model accuracy and robustness [25]. To fully exploit informative spectra, scientists have developed various variable selection algorithms, including interval partial least squares regression (iPLS) [26], backward interval partial least squares regression (biPLS) [27], moving-window partial least squares regression (MWPLS) [28], uninformative variable elimination (UVE) [29], successive projections algorithm (SPA) [30], and interval combination optimization (ICO) [31]. For instance, NIR combined with ICO was used to establish models for the four major components (total sugars, reducing sugars, total nitrogen, and nicotine) of tobacco, and exhibited a higher prediction accuracy compared to full-spectrum models [32]. Additionally, there is a growing approach toward developing handheld near-infrared spectroscopy devices to be easily applied for in situ determinations. NIR instruments are becoming increasingly smaller in size, less costly, more robust, and operable without specialized training for the operators. Portable NIR spectrometers are poised to broaden the application scope of NIR analytical techniques, thereby contributing significantly to grain inspection, food safety, and other related fields [33]. A portable NIR was used to collect rice spectra within the range of 800–1100 nm and MWPLS was applied to construct an analysis model for amylose content. The results demonstrated good accuracy and stability, with an R^2^ of 0.96 and an RMSEP of 0.4–0.5%. Furthermore, this model has been piloted in a cereal milling plant [34]. These variable selection algorithms each have unique mechanisms, advantages, and disadvantages in variable selection. In practical applications, the appropriate algorithm or combination of algorithms should be selected based on the characteristics of the spectral data and the analysis goals.

In this study, to enhance the universality and reliability of the near-infrared analysis model, 75 samples of TNs from multiple varieties and regions were collected, and portable NIR was utilized to improve the practicality. Additionally, in order to boost the predictive performance of the model, various preprocessing techniques and variable selection algorithms were employed. Subsequently, quantitative prediction models for crude oil (CO), crude protein (CP), and total starch (TS) were established. Ultimately, the prediction accuracy of the models was verified using unknown samples.

## 2. Materials and Methods

### 2.1. Sample Preparation and Near-Infrared Spectrum Collection

In the period from 2022 to 2023, we collected a total of 75 TN tuber samples of 28 varieties (Table S1). After washing, the fresh samples were air-dried outdoors for a week to maintain a moisture content of approximately 10%, then crushed and sieved through a 30-mesh screen. The processed samples were stored in a refrigerator at 4 °C.

The near-infrared spectra of the 75 TN samples were collected using a handheld near-infrared spectrometer (IAS8120, Intelligent Analysis Service Co., Ltd., Wuxi, China). Please see Figure S1 for an image of the instrument. Prior to acquiring the spectral data, the near-infrared spectrometer recorded a white reference spectrum and re-recorded it every 30 min. The 15.0 g TN powder samples were placed in a dedicated sample cup positioned at the light source of the spectrometer. The instrument collected spectra via diffuse reflection with a scanning wavelength range of 900–1700 nm, a resolution of 12 nm, and 20 scans per sample. Each sample was measured three times, and the original spectra were averaged to obtain a mean spectrum for subsequent modeling and analysis. The instrument automatically converts reflectivity to absorbance using the formula A = −log(R), where A represents absorbance and R represents reflectivity.

### 2.2. The Analyses of Crude Oil, Crude Protein, and Total Starch

Drawing upon the research of Duyi with minor modifications, the CO content of TNs was analyzed using the Soxhlet extraction method [6], with each sample being measured in triplicate for accuracy. A powder sample of 0.5 g was placed in an oven at 80 °C for 2 h to determine its dry weight. Subsequently, the samples were placed in fat-free filter paper thimbles and extracted with petroleum ether (boiling range: 30–60 °C) for 6 h. During the extraction, the petroleum ether was refluxed once every 10–15 min until it became clear and transparent. After extraction, the samples were dried for 30 min, and their dry weight was measured to calculate the crude fat content.

Drawing upon the research of Duyi with minor modifications, the Kjeldahl method was employed to measure the CP content of TNs [6], with each sample being measured in duplicate for accuracy. A 0.5 g sample was weighed into a digestion tube, and 10 milliliters of concentrated sulfuric acid was slowly added. The samples were then digested in a digestion furnace at 250 °C for 30 min, followed by an increase to 350 °C for another 30 min, and finally to 400 °C for 4 h. After cooling, the digest was transferred to a 250 milliliter volumetric flask and diluted to the mark. The nitrogen content was then determined using an automatic Kjeldahl nitrogen analyzer. The crude protein content was calculated by multiplying the nitrogen content by a conversion factor of 6.25.

The TS content was measured using a Total Starch Content Assay Kit (AKSU015, Boxbio, Beijing, China), with each sample being measured in triplicate for accuracy. The detection principle employed is based on the anthrone colorimetric assay [35]. Briefly, 50 mg of sample was weighed into a centrifuge tube and mixed thoroughly with 1 mL of elution buffer. The mixture was then incubated in a sealed water bath at 80 °C for 30 min to prevent water loss. Following incubation, the mixture was centrifuged at 8000 g for 10 min at room temperature. The supernatant was discarded, and the precipitate was retained. The precipitate was thoroughly mixed with 500 μL of distilled water and gelatinized in a water bath at 95 °C for 15 min. After cooling to room temperature, 1 mL of extraction buffer was added and thoroughly mixed. The mixture was then extracted at room temperature for 15 min, with shaking occurring 3–5 times during this period. Following extraction, the mixture was centrifuged at 8000 g for 10 min at room temperature, and the supernatant was collected for measurement. The collected supernatant was appropriately diluted, and the color development reagent was then added. The mixture was incubated at 95 °C for 10 min, cooled to room temperature, and the absorbance at 620 nm was measured to calculate the starch content.

### 2.3. Algorithms

During the spectral collection process, the influence of instrument noise or light scattering is encountered. Therefore, three preprocessing methods were specifically selected in this research. Savitzky–Golay smoothing (S–G smoothing) [36] is a digital filtering technique utilized for data smoothing and derivative calculation. It can reduce noise while preserving the characteristics of the signal as much as possible. In this study, the size of the smoothing window was set to 5 data points. Standard Normal Variate (SNV) [37] and Multiplicative Scatter Correction (MSC) [38] are primarily used to correct scattering effects in spectral data and are particularly applicable to mitigating the interference of light scattering caused by powder samples.

In addition, in order to remove irrelevant or redundant variables and improve the model performance, four kinds of variable selection algorithms were employed in this study: moving-window partial least squares regression (MWPLS) [28], interval partial least squares regression (iPLS) [26], interval combination optimization (ICO) [31], and uninformative variable elimination combined with the successive projections algorithm (UVE-SPA) [29,39]. Among them, MWPLS and iPLS are interval variable selection algorithms designed to identify specific intervals that are most correlated with model performance. In this study, for iPLS, we divided the entire spectral region into 10 equal parts to determine the optimal spectral region for the model. For MWPLS, the size of the moving window was set to 1/10 of the total number of variables in each iteration to identify the optimal spectral region. ICO is also an interval variable selection algorithm, but unlike the aforementioned two algorithms, it considers the relationship between combinations of intervals and the predicted values, seeking the most relevant combination of intervals. The procedure of ICO is outlined as follows: firstly, the spectra were generated into 40 equal-width intervals and 4000 random combinations via weighted bootstrap sampling. Secondly, the weight of each interval was calculated by the frequency of appearance. Thirdly, the PLS algorithm and five-fold cross-validation were used to extract the ratio of optimal intervals with lower RMSECVs. Finally, the intervals with lower RMSECVs were selected as the optimal intervals. The UVE-SPA is a variable selection method that combines the UVE algorithm and SPA. The main goal of the UVE algorithm is to reduce the number of variables included in the final model, thereby decreasing model complexity and improving model performance. The SPA selects wavelengths through projection analysis, enabling the selection of variable combinations with minimal redundant information and collinearity. Thus, the UVE-SPA can effectively reduce model complexity and enhance predictive accuracy. In the UVE algorithm section of this study, PLSR models were established using the spectral data and chemical values from the training set, with five-fold cross-validation employed. The maximum number of latent variables was set to 10, and information from all 10 components was utilized to eliminate variables that did not contribute any relevant information to the chemical values. For the SPA section, the default minimum number of selected variables was set to 1, and the maximum number was set to one-third of the number of variables selected by the UVE algorithm.

### 2.4. Software and Datasets

Quantitative analysis models were established for the near-infrared spectral data of TNs, using CO, CP, and TS as the analytical indicators. Each sample was measured three times using NIR, and the average of the three spectra was taken as the representative spectrum for that sample. Additionally, for each indicator, the values were ranked. Subsequently, systematic sampling (with a distance of 3) was used to divide the dataset into a calibration set (50 samples) and a validation set (25 samples). The model performance was optimized using 10-fold cross-validation. Data preprocessing, data modeling, and model plotting were all conducted using MATLAB software (Version R2024b, MathWorks, United States).

### 2.5. Evaluation Metrics

The model evaluation metrics utilized were the R^2^, RMSE of cross-validation (RMSECV), and RMSEP. A value of R^2^ closer to 1 and a smaller RMSEP indicate a stronger predictive performance of the model.

$$R^{2}=1-\frac{\sum {\left(y_{i}-{\hat{y}}_{i}\right)}^{2}}{\sum {\left(y_{i}-{\bar{y}}_{i}\right)}^{2}}$$

$$RMSE=\sqrt{\frac{1}{n}\sum_{i=1}^{n}{\left(y_{i}-{\hat{y}}_{i}\right)}^{2}}$$

where ${\;y}_{i}$ represents the actual value of the *i* observation, ${\hat{y}}_{i}$ represents the predicted value of the *i* observation, $\bar{y}$ represents the mean of the observed values, and *n* represents the total number of observations.

## 3. Results

### 3.1. Sample Composition Content

To ensure that the samples are representative, TNs of different varieties were collected from various provinces, implying a certain degree of variability in their chemical values. This variability ensures the generalization ability and robustness of the model. In this research, for the 75 collected samples, the values for CO, CP, and TS ranged from 8.45 to 26.83, 4.31 to 12.13, and 20.85 to 40.40, respectively. The sampling method employed was systematic sampling after ranking, which ensured that both the maximum and minimum values were included in the calibration set, thereby guaranteeing the representativeness of the modeling samples. The distribution of various indicators in each dataset of TN samples is displayed in Table 1.

### 3.2. Near-Infrared Spectroscopy and Preprocessing

**Figure 1:**
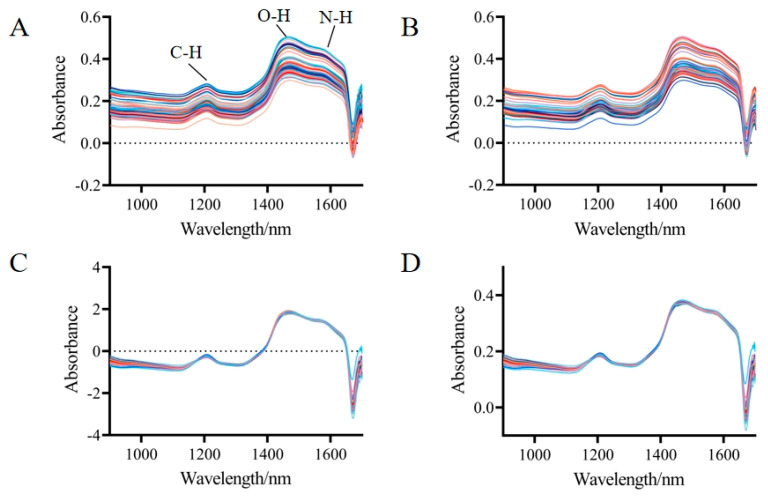
Near-infrared spectra of TNs. (**A**) Raw spectra. (**B**) Savitzky–Golay smoothing (S–G smoothing). (**C**) Standard Normal Variate (SNV). (**D**) Multiplicative Scatter Correction (MSC).

The sample spectra, which were extracted from TN samples within the wavelength range of 900–1700 nm, are shown in Figure 1A. The spectral curves of the different samples exhibited a consistent trend with nearly identical positions of the absorption peaks, indicating a high degree of similarity in the internal chemical composition of the samples. The differences in absorbance magnitudes could be attributed to variations in the content of individual compounds, as well as to physical effects such as scattering. The spectra primarily contained three characteristic peaks: the second overtone vibrational absorption of C-H at 1200 nm, the first overtone vibrational absorption of O-H at 1460 nm, and the first overtone vibrational absorption of N-H at 1580 nm. Besides containing the chemical information of the sample itself, the spectra also encompassed other irrelevant information, such as electrical noise, sample background, and stray light. In this study, three preprocessing methods (S–G smoothing, SNV, and MSC) were employed to optimize the spectra, as displayed in Figure 1B–D. After the preprocessing was completed, it could be seen solely from the spectrogram that both SNV and MSC had significantly reduced the spectral differences caused by light scattering.

### 3.3. Full-Spectrum Model

**Figure 2:**
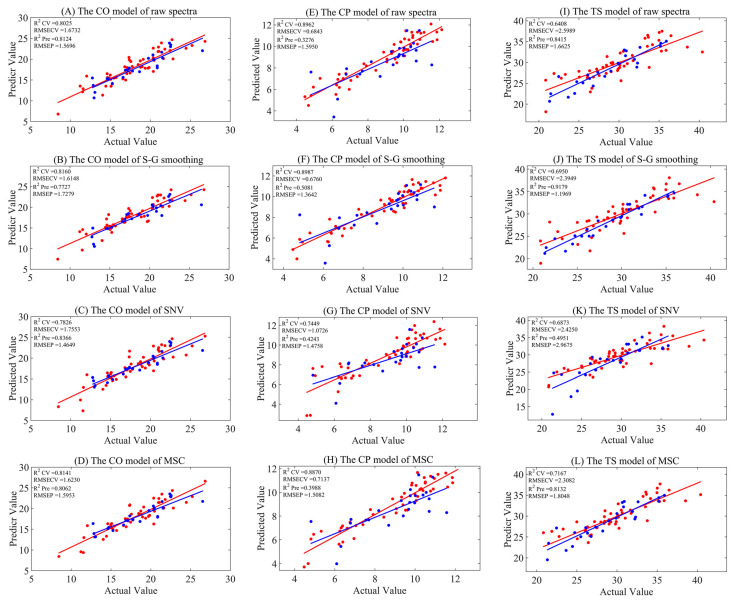
The model results of full-spectrum analysis combined with three preprocessing methods, where (**A**–**D**) represent CO models for raw, S–G smoothing, SNV, and MSC, respectively. (**E**–**H**) are CP models for raw, S–G smoothing, SNV, and MSC, respectively. (**I**–**L**) are TS models for raw, S–G smoothing, SNV, and MSC, respectively. Red represents the training set, and blue represents the test set.

Near-infrared models for crude oil (CO), crude protein (CP), and total starch (TS) were established using PLSR. The maximum number of latent variables (LVs) was limited to ten, and the optimal number of LVs was determined by the five-fold cross-validation technique. The models based on raw spectra and those processed with three different preprocessing methods are presented in Table 2 and Figure 2. For the raw spectra models, the R_CV_^2^ and R_Pre_^2^ values for CO, CP, and TS were 0.8025 and 0.8124; 0.8962 and 0.3276; and 0.6408 and 0.8415, respectively. Although the model for CO achieved a certain degree of prediction accuracy, there were issues of overfitting and underfitting in the model performance for CP and TS. After applying the three preprocessing methods, smoothing and SNV did not show significant improvement for the three indicators. However, the data preprocessed with MSC showed a substantial enhancement for TS, with R_CV_^2^ and R_Pre_^2^ values of 0.7167 and 0.8132, respectively, and maintained the model performance for CO, with R_CV_^2^ and R_Pre_^2^ values of 0.8141 and 0.8062, respectively. The results from the aforementioned preprocessing indicated that, although preprocessing could somewhat improve the predictive performance of a model for a certain indicator, the overall improvement was not obvious. In light of this, we adopted four different variable selection algorithms, aiming to explore and construct models with better performances.

### 3.4. Variable Selection Algorithm Model

The results of full-spectrum modeling in this study failed to meet the requirements of practical applications, as too many irrelevant variables caused a decrease in model accuracy. To address this issue, we next employed four variable selection algorithms to screen the spectral ranges suitable for quantitative near-infrared spectroscopy models.

#### 3.4.1. Variable Selection Model for CO

**Figure 3:**
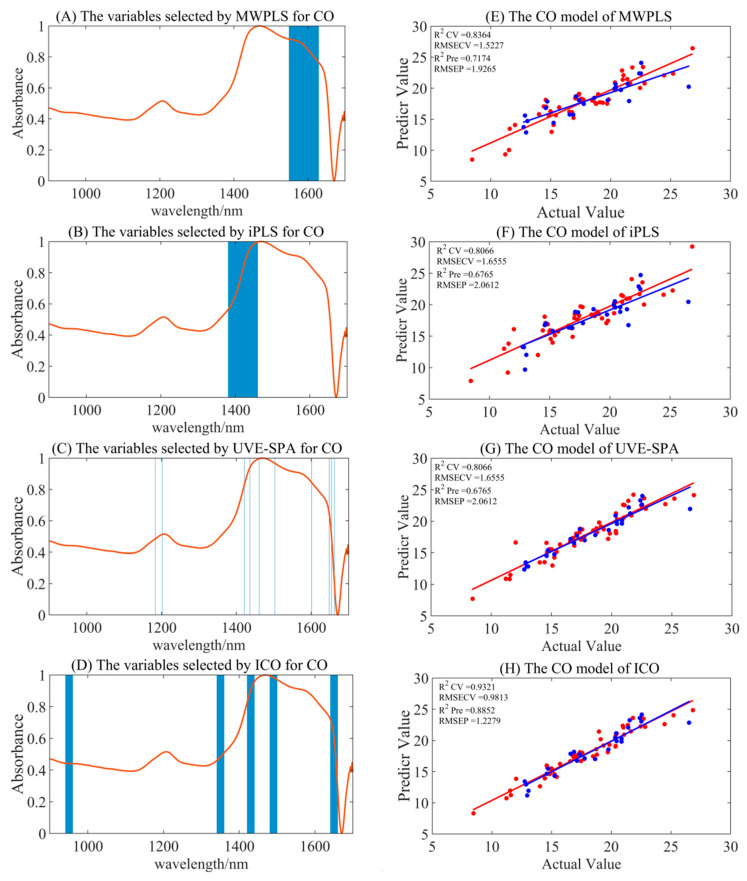
The summary of the optimal model results for CO under different variable selection algorithms. (**A**–**D**) are the selected regions of MWPLS, iPLS, UVE-SPA, and ICO, respectively. (**E**–**H**) represent the model results by utilizing the corresponding selected regions. Red represents the training set, and blue represents the test set.

Compared to the full-spectrum model for CO, the combination of S–G smoothing preprocessing with ICO, along with UVE-SPA variable selection models, demonstrated superior model accuracy, with R_pre_^2^ values of 0.8852 and 0.8946, and RMSEP values of 1.2279 and 1.1764. The detailed performances of variable selection algorithms are shown in Table 3 and Figure 3. However, the MWPLS and iPLS models did not contribute to optimization in this context. Interestingly, after SNV and MSC preprocessing, the ICO calibration set models generally exhibited better accuracy, but the performance of the validation set models declined, with R_pre_^2^ values of 0.8177 and 0.8473, and RMSEP values of 1.5471 and 1.5297, respectively. This might be attributed to the fact that SNV and MSC preprocessing highlighted more characteristic variables, leading to the overfitting of the models, which in turn resulted in poorer performance in the validation set.

The optimal preprocessing-screened feature variables from the two variable selection methods overlapped. In Figure 2, UVE-SPA variable selection yielded the following wavelengths: 1183, 1202, 1421, 1436, 1461, 1503, 1601, 1648, 1654, and 1662 nm. Meanwhile, ICO variable selection identified the following spectral ranges: 941–960, 1341–1360, 1421–1440, 1481–1500, and 1641–1660 nm. These selected spectral bands primarily reflect the characteristic absorption peaks of C-H bond overtones and combination bands, which correspond to the vibrational absorption of numerous -CH_2_ and -CH_3_ groups in CO. The chosen spectral bands possessed certain chemical interpretability and provided the necessary feature information for the model.

#### 3.4.2. Variable Selection Model for CP

**Figure 4:**
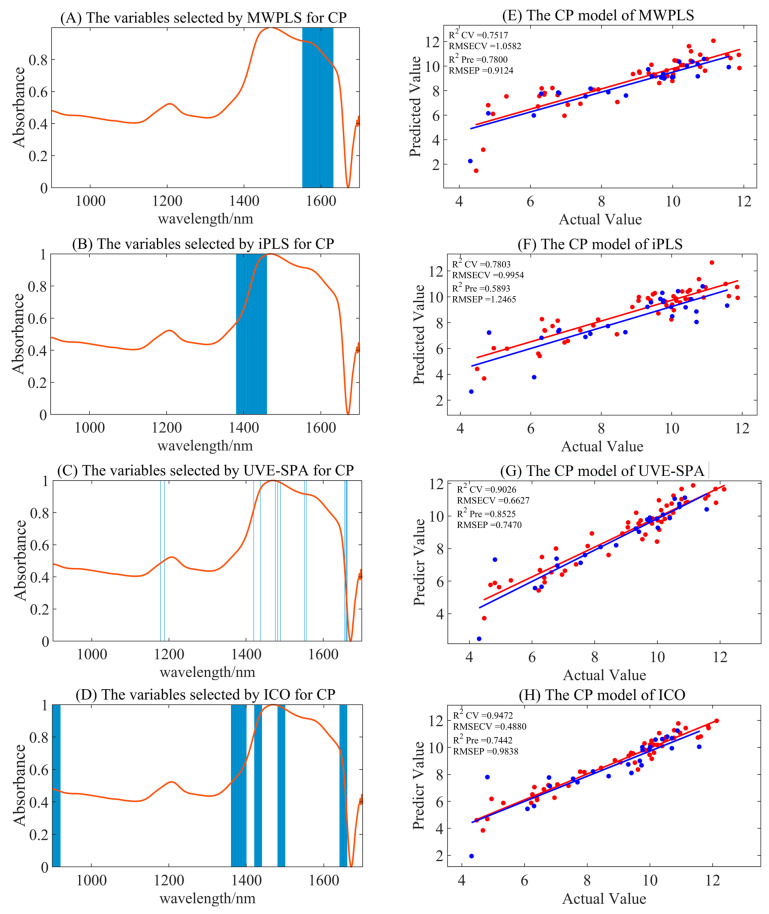
The summary of optimal model results for CP under different variable selection algorithms. (**A**–**D**) are the selected regions of MWPLS, iPLS, UVE-SPA, and ICO, respectively. (**E**–**H**) represent the model results by utilizing the corresponding selected regions. Red represents the training set, and blue represents the test set.

The full-spectrum model for CP exhibited significant overfitting, rendering the results impractical. After preprocessing and combining with four variable selection algorithms for modeling (displayed in Table 4 and Figure 4), only the combination of S–G smoothing with the UVE-SPA, and MSC preprocessing with MWPLS yielded reliable modeling results, with R_pre_^2^ values of 0.8525 and 0.7800, and RMSEP values of 0.7470 and 0.9124. The S–G smoothing combined with the UVE-SPA emerged as the optimal model, significantly enhancing model accuracy and stability. The ICO calibration set models generally demonstrated better accuracy, but the performance of the validation set models declined, indicating overall overfitting.

In Figure 4, UVE-SPA variable selection identified characteristic wavelengths at 1178, 1189, 1420, 1437, 1476, 1482, 1489, 1552, 1556, 1656, 1659, 1661, and 1662 nm, while the MWPLS variable selection yielded the range of 1552–1632 nm. These selections correspond to the absorption peaks of N-H, C-H, and O-H bonds, and to the abundant NH_3_, C-H, and -COOH groups in proteins. The selected variable region had a certain degree of chemical interpretability, which might be one of the reasons for the improved model performance after variable selection.

#### 3.4.3. Variable Selection Model of TS

**Figure 5:**
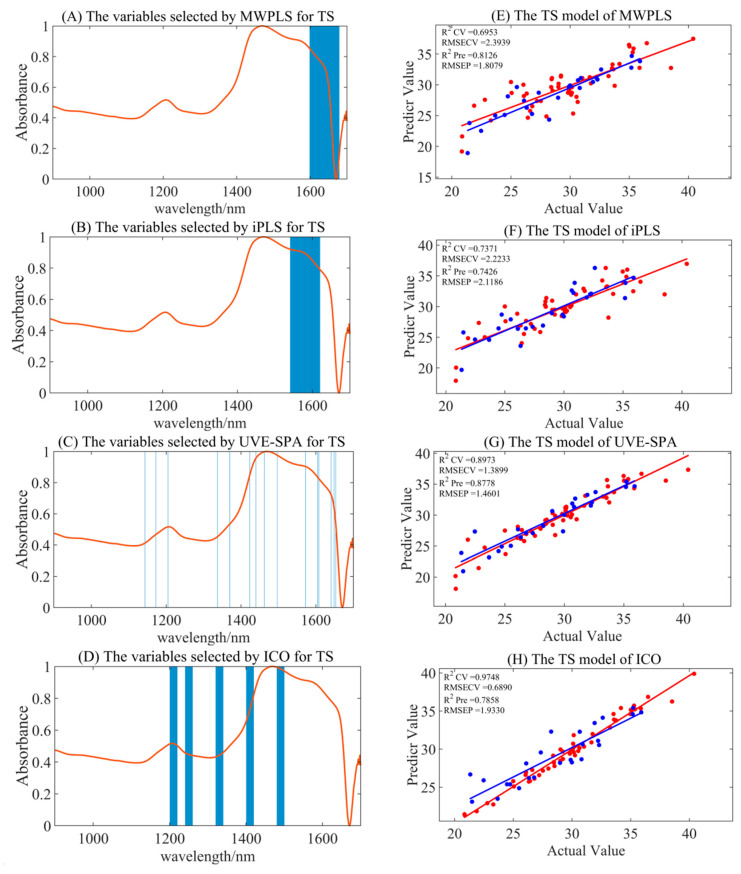
The summary of optimal model results for TS under different variable selection algorithms. (**A**–**D**) are the selected regions of MWPLS, iPLS, UVE-SPA, and ICO, respectively. (**E**–**H**) represent the model results by utilizing the corresponding selected regions. Red represents the trainging set, and blue represents the test set.

Compared to the full-spectrum model for TS, the MWPLS, iPLS, and UVE-SPA models significantly reduced the degree of overfitting, while the ICO model still exhibited overfitting, as presented in Table 5 and Figure 5. The optimal preprocessing methods for the MWPLS, iPLS, and UVE-SPA models were S–G smoothing, MSC, and MSC, respectively. The corresponding R_pre_^2^ values were 0.8126, 0.7426, and 0.8778, while the RMSEP values were 1.8079, 2.1186, and 1.460, respectively. The UVE-SPA model demonstrated the best accuracy and stability, while also reducing computational load, making it more suitable for portable NIR applications.

In Figure 5, the MWPLS variable selection yielded the range of 1599–1679 nm, the iPLS variable selection yielded the range of 1541–1620 nm, and the UVE-SPA identified specific wavelengths at 1143, 1172, 1205, 1337, 1370, 1423, 1440, 1462, 1497, 1572, 1605, 1608, 1641, 1649, and 1653 nm. These selections primarily focused on the characteristic absorption peaks of C-H and O-H bonds, which correspond to the abundant carbon–hydrogen bonds and hydroxyl groups on the glucose carbon chain. This demonstrated that the selected spectral bands had certain chemical interpretability and could significantly improve the stability of the model.

### 3.5. Comparison of Variable Selection and Full-Spectrum Modeling

**Figure 6:**
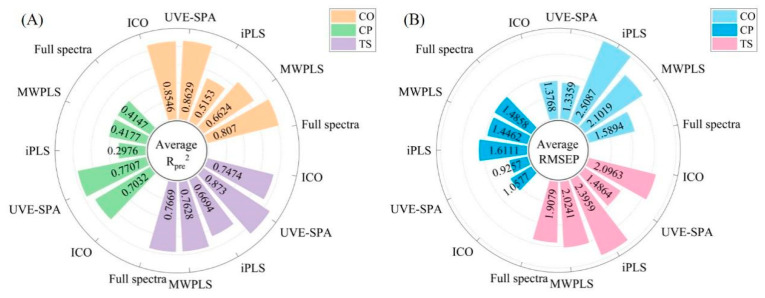
Comparison of the R_pre_^2^ and RMSEP values by variable selection and full-spectrum PLS. (**A**) is R_pre_^2^. (**B**) is RMSEP.

The three quality indicators each have their own unique characteristics. As shown in Figure 6, the CO model exhibited the best performance. The full-spectrum model had an average R_pre_^2^ of 0.8366 and an average RMSEP of 1.4649. Among the variable selection algorithms, the UVE-SPA was the most suitable, with the model achieving an average R_pre_^2^ of 0.8946 and an average RMSEP of 1.1764. Setting the interval to one-tenth of the full spectrum for both MWPLS and iPLS did not result in any optimization, which might indicate that the characteristic bands for CO were distributed across multiple regions within the spectrum. Therefore, the UVE-SPA was found to be more suitable.

The variable selection effect for CP was the most notable. The full-spectrum model had an average R_pre_^2^ of 0.5081 and an average RMSEP of 1.3642, whereas the most suitable variable selection algorithm was the UVE-SPA, resulting in a model with an average R_pre_^2^ of 0.8525 and an average RMSEP of 0.7470. The UVE-SPA not only significantly improves the accuracy of model predictions but also reduces model overfitting by selecting relevant variables, thus providing a reliable variable screening method for CP modeling.

For TS, the full-spectrum model exhibited an average R_pre_^2^ of 0.8132 and an average RMSEP of 1.8048. Among the variable selection algorithms, the UVE-SPA was the most appropriate, resulting in a model with an average Rpre^2^ of 0.8778 and an average RMSEP of 1.4601. The UVE-SPA significantly enhanced the stability of the model compared to the full-spectrum model, markedly reducing both overfitting and underfitting, and thus demonstrated greater practicality.

In the full-spectrum modeling, 801 variables were utilized, but the four different variable selection algorithms significantly decreased the number of variables involved in the modeling process. Among them, the UVE-SPA employed the smallest number of variables for modeling and achieved the highest model accuracy and stability. As illustrated in Figure 6, based on the evaluation metric RMSEP, the UVE-SPA clearly outperformed the full-spectrum model. Furthermore, the Rpre^2^ values of the full-spectrum models for all three indicators were not as satisfactory as those obtained with the UVE-SPA. Evidently, the UVE-SPA was the most suitable variable selection algorithm in this study, particularly for the CP and TS indicators, where the full-spectrum modeling performance was relatively poorer.

## 4. Discussion

In this study, four variable selection algorithms were utilized to extract the characteristic wavelength bands for the determination of crude oil, crude protein, and total starch in TNs. The performance of these algorithms was compared with that of a PLS full-spectrum model, revealing that UVE-SPA variable selection significantly optimized the model’s effectiveness. The optimal models for CO, CP, and TS exhibited R^2^ values of 0.8946, 0.8525, and 0.8778, with RMSEP values of 1.1764, 0.7470, and 1.4601, respectively. The absolute errors between the predicted and actual values for the three-indicator spectral measurements were 0.80, 0.59, and 0.99 (Table S2). The overall results indicated that variable selection holds considerable potential for enhancing the performance of PLS full-spectrum models.

Furthermore, there exist notable differences in the results obtained from different variable selection models. NIR combined with ICO was used to establish models for the four major components (total sugars, reducing sugars, total nitrogen, and nicotine) of tobacco, resulting in R^2^ values of 0.985, 0.9408, 0.937, 0.9648, respectively, and RMSEP values of 0.8672, 1.4464, 0.0432, 0.1078, respectively [29]. Additionally, MWPLS was applied to construct an analysis model for amylose content. The results demonstrated good accuracy and stability, with an R^2^ of 0.96 and an RMSEP of 0.4–0.5% [30]. However, in this study, the roles of both methods were limited and did not achieve the expected effects. We believe that the information distribution of the three indicators spans multiple spectral bands, and the selection of a single band limits the model’s ability to acquire characteristic information. A prediction model for soluble solids in Hami melons was established using MC-UVE-SPA, with correlation coefficients ranging from 0.81 to 0.93 and RMSEP values between 0.95 and 0.99 [40]. While UVE-SPA variable selection significantly optimized the prediction model for TNs, there is still room for improvement compared to near-infrared prediction models for major crops such as rice. Deep learning demonstrates stronger feature selection capabilities compared to machine learning. Zhangchu et al. utilized convolutional neural networks for feature selection and established near-infrared prediction models for total anthocyanin content, total flavonoids, and total phenolics in dried black goji berries [41]. Deep learning is well suited for large datasets, and as the number of samples accumulates, the enormous potential of deep learning as a method for modeling and feature extraction in regression problems can be anticipated.

Previous studies have reported promising results in predicting crude fat, crude protein, and total starch using NIRS in different materials. Yakubu A. B. et al. established NIRS prediction models for soybean oil and protein using PLS, achieving R^2^ values of 0.992 and 0.995, respectively, with corresponding RMSEP values of 0.208 and 0.280 [19]. Özcan Çataltas et al. utilized a one-dimensional convolutional autoencoder combined with NIRS to detect protein, starch, and oil content in corn kernels, obtaining R^2^ values of 0.9012, 0.9359, and 0.9632, respectively, with corresponding RMSEP values of 0.1535, 0.2093, and 0.0388. However, in another dataset reported in the same study, the R^2^ values for the protein, starch, and oil models were 0.8995, 0.7988, and 0.8199, respectively, with corresponding RMSEP values of 0.1548, 0.3709, and 0.0857 [15]. These R^2^ values are comparable to those of our model, but the RMSEP values are smaller. RMSEP is an indicator that measures the deviation between the predicted and actual values of a model. Given the similar R^2^ values, we hypothesize that the differences in errors between the traditional detection methods used for the two crops may be the cause. The detection system for soybeans is more developed. To obtain a more accurate NIRS model for TNs, we must continue to optimize the traditional detection methods for this crop to stabilize the reference values.

## 5. Conclusions

In this study, to achieve the rapid detection of the three major nutritional components in TNs, a portable near-infrared spectrometer, combined with chemometrics, was used to establish rapid prediction models for these nutrients. Among these models, the one using full-spectrum data combined with three preprocessing methods exhibited poor performance. To enhance the performance of the model, four variable selection algorithms were employed to construct near-infrared analysis models for CO, CP, and TS in TNs. The results demonstrated that the models built using the UVE-SPA for all three indicators surpassed the full-spectrum models, making the UVE-SPA the most suitable variable selection algorithm for TN analysis. Especially for CP and TS, the characteristic wavelengths selected by the UVE-SPA directly reflected the correspondence between sample composition and spectral information. The UVE-SPA eliminated non-informative variables, thereby enhancing the predictive capability of the model and significantly reducing its computational load.

This study developed near-infrared non-destructive analysis models for three conventional evaluation components of TNs and improved the predictive and generalization abilities of the models through the use of variable selection algorithms. After ensuring the predictive performance of the NIR model, it demonstrated significant advantages, such as rapidness (approximately 30 s per sample), simultaneous multi-component analysis, and environmental friendliness (generating no exhaust gases or liquid waste), and provided new technologies and methods for TN quality assessment, determination of the optimal harvest time, and graded sales. In addition, in our study, we modeled the data by grinding TNs into powder. In the future, to further accelerate the rapid detection of the nutritional components in TNs, we could consider directly detecting the intact oil shale and establishing a mathematical prediction model.

## Figures and Tables

**Table 1:** The distribution of various indicators in each dataset of TN samples.

Indicators	Datasets	Numbers	Max Value	Min Value	Mean Value
CO	Calibration set	50	26.83	8.45	17.95
	Validation set	25	26.51	12.75	18.40
CP	Calibration set	50	12.13	4.31	8.90
	Validation set	25	11.57	4.47	8.79
TS	Calibration set	50	40.40	20.85	29.82
	Validation set	25	35.90	21.33	28.60

Notes: CO, crude oil; CP, crude protein; and TS, total starch.

**Table 2:** Results of full-spectrum analysis models for three components.

Preprocessing Methods	Indicators	nLV	R_cv_^2^	RMSECV	R_pre_^2^	RMSEP
Raw spectra	CO	10	0.8025	1.6732	0.8124	1.5696
	CP	10	0.8962	0.6843	0.3276	1.5950
	TS	10	0.6408	2.5989	0.8415	1.6625
S–G smoothing	CO	7	0.8160	1.6148	0.7727	1.7279
	**CP**	**10**	**0.8987**	**0.6760**	**0.5081**	**1.3642**
	TS	10	0.6950	2.3949	0.9179	1.1969
SNV	**CO**	**10**	**0.7826**	**1.7553**	**0.8366**	**1.4649**
	CP	7	0.7449	1.0726	0.4243	1.4758
	TS	3	0.6873	2.4250	0.4951	2.9675
MSC	CO	10	0.8141	1.6230	0.8062	1.5953
	CP	10	0.8870	0.7137	0.3988	1.5082
	**TS**	**10**	**0.7167**	**2.3082**	**0.8132**	**1.8048**

Notes: nLV, number of latent variables; RMSECV, root mean square error of cross-validation; R_cv_^2^, correlation coefficient of cross-validation; RMSEP, root mean square error of prediction; and Rpre^2^, correlation coefficient of prediction; bold indicates the optimal model.

**Table 3:** Results of variable selection analysis models for CO.

Preprocessing Methods	Variable Selection Algorithms	nLV	R_cv_^2^	RMSECV	R_pre_^2^	RMSEP
Raw spectra	MWPLS	5	0.7716	1.7991	0.6275	2.2117
	iPLS	3	0.6885	2.1013	0.5088	2.5399
	UVE-SPA	9	0.8700	1.3575	0.8677	1.3182
	ICO	10	0.9383	0.9355	0.8682	1.3157
S–G smoothing	MWPLS	5	0.7747	1.7870	0.6220	2.2279
	iPLS	5	0.7160	2.0062	0.4534	2.6792
	UVE-SPA	**10**	**0.8718**	**1.3478**	**0.8946**	**1.1764**
	ICO	**10**	**0.9321**	**0.9813**	**0.8852**	**1.2279**
SNV	MWPLS	4	0.7778	1.7746	0.6826	2.0416
	iPLS	3	0.6466	2.2380	0.4223	2.7544
	UVE-SPA	10	0.8716	1.3491	0.8674	1.3194
	ICO	10	0.9540	0.8077	0.8177	1.5471
MSC	MWPLS	**4**	**0.8364**	**1.5227**	**0.7174**	**1.9265**
	iPLS	**5**	**0.8066**	**1.6555**	**0.6765**	**2.0612**
	UVE-SPA	10	0.8938	1.2268	0.8218	1.5297
	ICO	10	0.9594	0.7589	0.8473	1.4163

Notes: Bold indicates the optimal model.

**Table 4:** Results of variable selection analysis models for CP.

Pretreatment Methods	Variable Selection Techniques	nLV	R_cv_^2^	RMSECV	R_pre_^2^	RMSEP
Raw spectra	MWPLS	3	0.7313	1.1008	0.1980	1.7419
	iPLS	4	0.7058	1.1518	0.0365	1.9092
	UVE-SPA	10	0.9313	0.5566	0.7454	0.9815
	ICO	10	0.9607	0.4208	0.6575	1.1383
S–G smoothing	MWPLS	3	0.7341	1.0951	0.2228	1.7148
	iPLS	3	0.7112	1.1412	0.1885	1.7522
	UVE-SPA	**9**	**0.9026**	**0.6627**	**0.8525**	**0.7470**
	ICO	10	0.9532	0.4594	0.6775	1.1045
SNV	MWPLS	5	0.6796	1.2020	0.4701	1.4159
	iPLS	2	0.7059	1.1517	0.3759	1.5366
	UVE-SPA	9	0.9260	0.5776	0.7418	0.9884
	ICO	**10**	**0.9472**	**0.4880**	**0.7442**	**0.9838**
MSC	MWPLS	**4**	**0.7517**	**1.0582**	**0.7800**	**0.9124**
	iPLS	**5**	**0.7803**	**0.9954**	**0.5893**	**1.2465**
	UVE-SPA	10	0.9446	0.5000	0.7432	0.9857
	ICO	10	0.9487	0.4810	0.7336	1.0040

Notes: Bold indicates the optimal model.

**Table 5:** Results of the variable selection analysis model for TS.

Pretreatment Methods	Variable Selection Techniques	nLV	R_cv_^2^	RMSECV	R_pre_^2^	RMSEP
Raw spectra	MWPLS	5	0.6927	2.4039	0.7977	1.8784
	iPLS	3	0.6561	2.5431	0.6473	2.4801
	UVE-SPA	10	0.7901	1.9870	0.8906	1.3813
	ICO	7	0.9523	0.9470	0.7139	2.2340
S–G smoothing	MWPLS	**5**	**0.6953**	**2.3939**	**0.8126**	**1.8079**
	iPLS	3	0.6600	2.5285	0.6479	2.4783
	UVE-SPA	10	0.7972	1.9528	0.8621	1.5508
	ICO	7	0.9513	0.9566	0.7345	2.1519
SNV	MWPLS	4	0.7902	1.9864	0.6922	2.3168
	iPLS	4	0.6161	2.6869	0.6398	2.5064
	UVE-SPA	7	0.9079	1.3160	0.8616	1.5535
	ICO	7	0.9648	0.8134	0.7553	2.0661
MSC	MWPLS	4	0.7675	2.0909	0.7488	2.0932
	iPLS	**4**	**0.7371**	**2.2233**	**0.7426**	**2.1186**
	UVE-SPA	**7**	**0.8973**	**1.3899**	**0.8778**	**1.4601**
	ICO	**7**	**0.9748**	**0.6890**	**0.7858**	**1.9330**

Notes: Bold indicates the optimal model.

## Data Availability

The original contributions presented in the study are included in the article/Supplementary Material, further inquiries can be directed to the corresponding authors.

## Supplementary Materials

The following supporting information can be downloaded at: https://www.mdpi.com/article/10.3390/foods14030366/s1, Figure S1. IAS8120 handheld near-infrared spectrometer; Table S1. information of TN samples; Table S2. Comparison between the predicted values of crude oil and the true values from analytical methods; Table S3. Comparison between the predicted values of crude protein and the true values from analytical methods; Table S4. Comparison between the predicted values of total starch and the true values from analytical methods.
