# Supplementary material for: The Application of Near-Infrared Spectroscopy Combined with Chemometrics in the Determination of the Nutrient Composition in Chinese Cyperus esculentus L
Source: Foods. 2025 Jan 23;14(3):366. doi: 10.3390/foods14030366 (PMC11817964; doi:10.3390/foods14030366)
Supplement: Supplementary file 1 [file foods-14-00366-s001.zip › foods-3399362-supplementary.pdf]

Application of Near-Infrared Spectroscopy Combined with Chemometrics in the Determination of Nutrient Composition in Chinese *Cyperus esculentus L.*

Xiaobo Jiao [1], Dongliang Guo [1], Xinjun Zhang [1], Yunpeng Su [1], Rong Ma [1], Lewen Chen [1], Kun Tian [1], Jingyu Su [1], Tangnuer Sahati [1], Xiahenazi Aierkenjiang [1], Jingjing Xia [1*] and Liqiong Xie [1,2*]

1 Xinjiang Key Laboratory of Biological Resources and Genetic Engineering, College of Life Science and Technology, Xinjiang University, Urumqi 830046, China.
2 College of Smart Agriculture, Xinjiang University, Urumqi 830046, China.
* Correspondence:

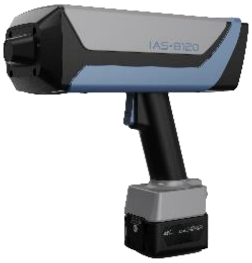

Fig.S1 IAS8120 handheld near-infrared spectrometer.

Table.S1 information of TN samples.

Serial Number	Variety	Year	Region
1	FC2	2022	Xinjiang Uyghur Autonomous Region Shache County
2	YYS1	2022	Henan Province
3	YYS2	2022	Henan Province
4	YYS3	2022	Henan Province
5	ZYS1	2022	Henan Province
6	ZYS5	2022	Henan Province
7	HD	2022	Henan Province
8	FC2	2022	Xinjiang Uyghur Autonomous Region Shache County
9	DL	2022	Xinjiang Uyghur Autonomous Region Shache County
10	FC2	2022	Xinjiang Uyghur Autonomous Region Shache County
11	CL1	2023	Xinjiang Uyghur Autonomous Region Shache County
12	CL2	2023	Xinjiang Uyghur Autonomous Region Shache County
13	CL3	2023	Xinjiang Uyghur Autonomous Region Shache County
14	JS8	2023	Xinjiang Uyghur Autonomous Region Shache County
15	JS9	2023	Xinjiang Uyghur Autonomous Region Shache County
16	JS12	2023	Xinjiang Uyghur Autonomous Region Shache County
17	JYD12	2023	Xinjiang Uyghur Autonomous Region Shache County

18	JS13	2023	Xinjiang Uyghur Autonomous Region Shache County
19	JS14	2023	Xinjiang Uyghur Autonomous Region Shache County
20	JYD14	2023	Xinjiang Uyghur Autonomous Region Shache County
21	JS15	2023	Xinjiang Uyghur Autonomous Region Shache County
22	JS18	2023	Xinjiang Uyghur Autonomous Region Shache County
23	JS21	2023	Xinjiang Uyghur Autonomous Region Shache County
24	JS23	2023	Xinjiang Uyghur Autonomous Region Shache County
25	JS24	2023	Xinjiang Uyghur Autonomous Region Shache County
26	JS25	2023	Xinjiang Uyghur Autonomous Region Shache County
27	JS26	2023	Xinjiang Uyghur Autonomous Region Shache County
28	JS38	2023	Xinjiang Uyghur Autonomous Region Shache County
29	JS8	2023	Xinjiang Uyghur Autonomous Region Fukang City
30	JS9	2023	Xinjiang Uyghur Autonomous Region Fukang City
31	JS10	2023	Xinjiang Uyghur Autonomous Region Fukang City
32	JS12	2023	Xinjiang Uyghur Autonomous Region Fukang City
33	JS13	2023	Xinjiang Uyghur Autonomous Region Fukang City
34	JS14	2023	Xinjiang Uyghur Autonomous Region Fukang City
35	JS15	2023	Xinjiang Uyghur Autonomous Region Fukang City
36	JS18	2023	Xinjiang Uyghur Autonomous Region Fukang City
37	JS21	2023	Xinjiang Uyghur Autonomous Region Fukang City
38	JS23	2023	Xinjiang Uyghur Autonomous Region Fukang City
39	JS24	2023	Xinjiang Uyghur Autonomous Region Fukang City
40	JS25	2023	Xinjiang Uyghur Autonomous Region Fukang City
41	JS26	2023	Xinjiang Uyghur Autonomous Region Fukang City
42	JS38	2023	Xinjiang Uyghur Autonomous Region Fukang City
43	JYD14	2023	Xinjiang Uyghur Autonomous Region Fukang City
44	CL2	2023	Xinjiang Uyghur Autonomous Region Fukang City
45	CL3	2023	Xinjiang Uyghur Autonomous Region Fukang City
46	Unknown	2023	Xinjiang Uyghur Autonomous Region Fukang City
47	Unknown	2023	Xinjiang Uyghur Autonomous Region Fukang City
48	Unknown	2023	Xinjiang Uyghur Autonomous Region Fukang City
49	Unknown	2023	Xinjiang Uyghur Autonomous Region Fukang City
50	Unknown	2023	Xinjiang Uyghur Autonomous Region Fukang City

51	Unknown	2023	Xinjiang Uyghur Autonomous Region Fukang City
52	Unknown	2023	Xinjiang Uyghur Autonomous Region Fukang City
53	Unknown	2023	Xinjiang Uyghur Autonomous Region Fukang City
54	Unknown	2023	Xinjiang Uyghur Autonomous Region Fukang City
55	Unknown	2023	Xinjiang Uyghur Autonomous Region Fukang City
56	Unknown	2023	Xinjiang Uyghur Autonomous Region Fukang City
57	Unknown	2023	Xinjiang Uyghur Autonomous Region Fukang City
58	Unknown	2023	Xinjiang Uyghur Autonomous Region Fukang City
59	Unknown	2023	Xinjiang Uyghur Autonomous Region Fukang City
60	Unknown	2023	Xinjiang Uyghur Autonomous Region Fukang City
61	FC2	2023	Xinjiang Uyghur Autonomous Region Shache County
62	FC2	2023	Xinjiang Uyghur Autonomous Region Shache County
63	FC2	2023	Xinjiang Uyghur Autonomous Region Shache County
64	FC2	2023	Xinjiang Uyghur Autonomous Region Shache County
65	FC2	2023	Xinjiang Uyghur Autonomous Region Shache County
66	FC2	2023	Xinjiang Uyghur Autonomous Region Shache County
67	FC2	2023	Xinjiang Uyghur Autonomous Region Shache County
68	FC2	2023	Xinjiang Uyghur Autonomous Region Shache County
69	FC2	2023	Xinjiang Uyghur Autonomous Region Shache County
70	FC2	2023	Xinjiang Uyghur Autonomous Region Shache County
71	FC2	2023	Xinjiang Uyghur Autonomous Region Shache County
72	FC2	2023	Xinjiang Uyghur Autonomous Region Shache County
73	FC2	2023	Xinjiang Uyghur Autonomous Region Shache County
74	FC2	2023	Xinjiang Uyghur Autonomous Region Shache County
75	FC2	2023	Xinjiang Uyghur Autonomous Region Shache County

Table.S2 Comparison between the predicted values of crude oil and the true values from analytical methods.

Calibration set	True value	Predicted value	Absolute error	Validation set	True value	Predicted value	Absolute error
1	26.83	24.07	2.76	1	22.55	24.01	1.46
2	25.21	23.54	1.67	2	26.51	22.05	4.46
3	22.70	23.60	0.90	3	20.41	20.02	0.39
4	20.96	22.58	1.62	4	20.85	19.66	1.20
5	21.07	22.52	1.45	5	22.52	22.62	0.10
6	20.37	18.16	2.21	6	20.32	20.92	0.60
7	21.81	24.22	2.41	7	17.12	17.70	0.58
8	21.01	20.64	0.37	8	22.39	23.31	0.92
9	20.36	21.23	0.87	9	20.45	19.62	0.83
10	11.59	11.40	0.19	10	20.86	20.10	0.76
11	20.46	20.28	0.18	11	19.76	18.67	1.08
12	12.03	16.69	4.66	12	12.86	13.46	0.60
13	24.46	22.75	1.71	13	18.64	17.86	0.79
14	19.11	19.12	0.02	14	21.42	22.13	0.71
15	21.64	21.04	0.60	15	16.88	16.51	0.36
16	18.79	18.16	0.62	16	16.58	17.10	0.52
17	17.08	17.74	0.66	17	17.76	17.01	0.75
18	22.45	22.53	0.08	18	12.75	12.56	0.19
19	19.34	18.71	0.63	19	17.36	18.76	1.40
20	21.39	23.20	1.81	20	15.24	14.62	0.63
21	22.83	21.85	0.99	21	21.54	21.03	0.51
22	17.05	18.09	1.04	22	12.95	12.89	0.07
23	20.35	18.43	1.92	23	14.59	14.50	0.09
24	18.74	18.87	0.14	24	14.72	15.38	0.66
25	17.56	18.73	1.16	25	13.06	12.82	0.24
26	16.79	17.34	0.55	**Average**	**18.40**	**18.21**	**0.80**
27	8.45	7.57	0.88				
28	17.32	18.07	0.75				
29	11.22	10.88	0.33				
30	19.71	17.32	2.39				
31	17.45	17.06	0.39				
32	16.90	16.04	0.86				
33	17.73	17.56	0.17				
34	19.85	18.19	1.66				
35	15.04	15.53	0.49				
36	15.71	16.29	0.58				
37	17.37	18.73	1.36				
38	18.96	19.74	0.78				
39	18.47	18.46	0.01				
40	16.56	16.71	0.15				
41	15.08	13.00	2.08				

42	14.98	15.52	0.54
43	15.43	15.21	0.22
44	14.59	16.57	1.98
45	15.24	14.23	1.01
46	14.43	13.52	0.91
47	11.53	11.02	0.51
48	14.85	15.62	0.77
49	14.64	15.06	0.42
50	14.03	13.44	0.59
**Average**	**17.95**	**17.94**	**1.02**

Table.S3 Comparison between the predicted values of crude protein and the true values from analytical methods.

Calibration set	True value	Predicted value	Absolute error	Validation set	True value	Predicted value	Absolute error
1	10.77	11.79	1.02	1	4.31	2.26	2.06
2	10.91	10.69	0.22	2	11.57	10.40	1.17
3	11.14	11.53	0.38	3	10.88	11.29	0.41
4	10.77	10.59	0.19	4	10.55	10.89	0.33
5	11.86	11.66	0.20	5	10.70	10.55	0.15
6	10.96	10.85	0.11	6	4.82	7.37	2.56
7	6.21	5.10	1.11	7	6.09	5.38	0.71
8	11.62	11.26	0.37	8	10.70	10.50	0.21
9	6.32	7.49	1.18	9	10.18	10.52	0.34
10	11.87	11.49	0.38	10	9.76	10.00	0.24
11	6.40	6.18	0.23	11	9.77	9.70	0.07
12	12.13	12.10	0.04	12	9.98	9.99	0.01
13	6.25	6.39	0.14	13	6.30	5.73	0.57
14	6.62	6.39	0.22	14	6.78	7.61	0.83
15	4.67	4.95	0.27	15	8.19	8.38	0.19
16	4.47	3.46	1.01	16	10.40	10.16	0.23
17	11.53	11.10	0.43	17	10.01	9.56	0.45
18	6.39	6.31	0.08	18	6.81	7.39	0.58
19	10.01	9.73	0.28	19	9.73	8.89	0.84
20	4.95	6.56	1.62	20	7.55	7.12	0.43
21	10.05	10.64	0.59	21	7.69	7.65	0.04
22	10.12	10.24	0.12	22	9.68	9.33	0.35
23	9.47	9.84	0.37	23	9.41	8.27	1.14
24	10.06	9.17	0.89	24	8.68	8.02	0.66
25	10.13	9.31	0.82	25	9.31	9.22	0.09
26	10.24	9.91	0.32	**Average**	**8.79**	**8.65**	**0.59**
27	7.05	6.92	0.13				
28	9.06	8.98	0.08				
29	9.52	9.21	0.31				

30	10.39	9.63	0.76
31	8.88	9.21	0.33
32	5.32	6.31	0.98
33	10.50	10.64	0.14
34	9.62	8.79	0.83
35	7.78	8.21	0.43
36	8.44	8.19	0.26
37	9.83	10.05	0.22
38	4.81	5.67	0.86
39	7.40	6.84	0.56
40	6.96	6.15	0.81
41	9.99	9.07	0.92
42	9.33	9.72	0.40
43	7.91	8.64	0.73
44	10.30	10.74	0.44
45	9.07	9.18	0.12
46	9.85	10.04	0.19
47	10.51	11.03	0.51
48	9.40	9.74	0.34
49	10.46	11.10	0.65
50	6.76	7.76	0.99
**Average**	**8.90**	**8.93**	**0.49**

Table.S4 Comparison between the predicted values of total starch and the true values from analytical methods.

Calibration set	True value	Predicted value	Absolute error	Validation set	True value	Predicted value	Absolute error
1	26.09	28.11	2.02	1	25.49	25.04	0.46
2	25.00	27.50	2.50	2	24.73	24.92	0.19
3	20.87	18.12	2.74	3	28.23	28.26	0.03
4	40.40	37.33	3.07	4	26.10	27.70	1.60
5	26.05	26.32	0.28	5	26.78	27.11	0.34
6	25.05	23.70	1.36	6	21.33	23.89	2.56
7	21.88	26.03	4.14	7	26.33	26.41	0.08
8	27.52	26.66	0.86	8	27.34	27.09	0.25
9	20.85	20.17	0.67	9	35.17	34.55	0.62
10	27.20	27.39	0.19	10	24.46	24.18	0.28
11	26.33	26.18	0.15	11	23.66	23.18	0.48
12	33.66	34.65	0.99	12	35.90	34.66	1.24
13	23.30	24.75	1.46	13	28.98	30.65	1.67
14	26.42	27.59	1.17	14	29.89	27.38	2.51
15	22.79	21.47	1.32	15	29.99	30.04	0.05
16	26.80	26.94	0.15	16	22.47	27.35	4.88
17	34.98	36.33	1.35	17	35.21	35.40	0.19

18	30.16	31.34	1.19
19	26.62	25.80	0.81
20	36.48	36.68	0.20
21	29.23	26.77	2.46
22	27.99	27.80	0.20
23	35.36	35.82	0.45
24	29.82	30.18	0.37
25	35.02	35.57	0.55
26	35.29	35.59	0.30
27	28.40	29.15	0.75
28	35.87	34.35	1.52
29	28.46	28.64	0.18
30	33.58	35.67	2.08
31	29.01	30.33	1.32
32	38.53	35.57	2.96
33	31.76	33.12	1.36
34	29.22	29.90	0.68
35	28.43	28.58	0.15
36	28.51	29.26	0.75
37	33.25	33.00	0.24
38	33.77	32.05	1.71
39	34.17	33.71	0.46
40	30.64	30.09	0.55
41	29.90	29.17	0.73
42	30.08	31.19	1.11
43	30.25	29.95	0.31
44	31.03	29.32	1.70
45	33.54	32.79	0.75
46	30.10	30.72	0.62
47	30.53	29.65	0.88
48	29.02	28.43	0.59
49	31.66	31.53	0.13
50	30.15	31.13	0.99
**Average**	**29.82**	**29.84**	**1.07**

18	30.81	31.33	0.52
19	31.93	33.27	1.34
20	32.62	33.72	1.10
21	30.91	32.67	1.76
22	21.49	20.96	0.53
23	32.31	32.07	0.24
24	32.22	31.54	0.68
25	30.66	31.88	1.21
**Average**	28.60	29.01	0.99
